# HIV Dolutegravir resistance and multiclass failure in Mozambique: findings from a real-world cohort

**DOI:** 10.1186/s12879-025-11639-2

**Published:** 2025-09-29

**Authors:** Fausto Ciccacci, Anna Maria Doro Altan, Noorjehan Majid, Stefano Orlando, Elton Uamusse, Marcia Rafael, Zita Sidumo, Paola Germano, Giovanni Guidotti, Carlo Federico Perno

**Affiliations:** 1 Department of Biomedicine and Prevention, University of Rome Tor Vergata, Rome, Italy; 2 Link Campus University, Rome, Italy; 3 DREAM Program, Community of Sant’Egidio, Maputo, Mozambique; 4 DREAM Program, Community of Sant’Egidio, Rome, Italy; 5 ASL Roma 1, Rome, Italy; 6 Bambino Gesù Pediatric Hospital IRCCS, Rome, Italy

**Keywords:** HIV drug resistance, Antiretroviral Therapy, Africa, Integrase strand transfer inhibitors

## Abstract

**Background:**

Dolutegravir (DTG) is the anchor drug of the first-line agent for HIV treatment globally, including low- and middle-income countries. Although clinical trials report low rates of integrase inhibitor resistance, real-world data from sub-Saharan Africa suggest a different scenario. We aimed to assess the prevalence and patterns of DTG resistance in Mozambique, building on prior preliminary findings with an expanded cohort and extended observation period.

**Methods:**

We conducted a retrospective observational study in five DREAM centers in Mozambique. HIV-positive individuals on DTG-based antiretroviral therapy (ART) with confirmed virological failure (HIV RNA > 1000 copies/mL) between July 2022 and December 2024 were included. Participants underwent genotypic resistance testing after six months of enhanced adherence support. Resistance mutations were identified via Sanger sequencing and interpreted using the Stanford HIV Drug Resistance Database.

**Results:**

Of 28 individual tested, 13 (46.4%) exhibited DTG resistance (12/13 with intermediate or high-level resistance); 92.3% harboured HIV-1 subtype C. Frequently observed mutations included G118R (43.8%), E138K (43.8%), L74M (31.3%), and R263K (25.0%), often in combination. All DTG-resistant individuals who underwent testing for other drug classes (*n* = 9) also showed co-resistance to NRTIs and/or NNRTIs. Notably, high-level resistance emerged also in 6 failing participants having shifted to DTG while virologically suppressed.

**Conclusions:**

This study reports the presence of DTG resistance and multiclass failure in Mozambique, suggesting potential limitations of current strategies. Sentinel surveillance and expanded access to resistance testing are important to preserve the efficacy of DTG-based regimens and inform future deployment of long-acting therapies in sub-Saharan Africa.

## Introduction

Despite major global advances in antiretroviral therapy (ART), HIV remains a significant public health challenge, with nearly 40 million people living with the virus worldwide as of 2023 [1]. The introduction of integrase strand transfer inhibitors (INSTIs) has transformed HIV treatment by offering potent, well-tolerated regimens with a high genetic barrier to resistance. Among these agents, dolutegravir (DTG) has become the preferred option for first-line ART globally, supported by robust evidence of efficacy, safety, and low rates of treatment-emergent resistance [2]. Following the 2018 endorsement by the World Health Organization (WHO), DTG has been rapidly adopted across low- and middle-income countries (LMICs), particularly in sub-Saharan Africa, where the burden of HIV is highest. By 2023, over 90% of LMICs had integrated DTG into their national guidelines, and large-scale roll-out was associated with significant improvements in viral suppression rates [3]. In Mozambique—one of the most affected countries, with an estimated 2.4 million people living with HIV—DTG was introduced as part of first-line therapy in 2019 and now represents the backbone of the national ART program [4].

However, growing evidence suggests that the real-world durability of DTG may be challenged in specific populations and contexts. Reports of resistance-associated mutations to DTG are emerging with increasing frequency, particularly in individuals with prior ART exposure, poor adherence, or delayed recognition of virological failure [3, 5, 6]. Mutations such as G118R, E138K, and R263K have been identified in multiple settings and are associated with reduced susceptibility to DTG and, in some cases, cross-resistance to other INSTIs [7]. These findings are especially concerning in regions with high prevalence of HIV-1 subtype C, where certain resistance pathways may be more accessible to the virus due to subtype-specific polymorphisms [8]. Yet, in countries such as Mozambique, real-world data on resistance patterns remain sparse, and the generalizability of findings from other regions is limited by differences in treatment histories, subtype prevalence, and health system infrastructure.

Although routine viral load monitoring has expanded in recent years, access to genotypic resistance testing remains limited in most LMICs. In Mozambique, surveillance data on DTG resistance are scarce, and little is known about the mutational patterns emerging in individuals who fail DTG-based therapy. In the absence of timely resistance surveillance, clinicians are often forced to make empirical decisions regarding regimen switches, which may lead to suboptimal outcomes or missed opportunities to contain emerging resistance. This knowledge gap hampers the timely identification of resistance, the optimization of salvage regimens, and the preservation of DTG’s long-term effectiveness. Elsewhere, we evaluated dolutegravir resistance in a small sample of individuals with virological failure in Mozambique, providing one of the first real-world signals of integrase inhibitor resistance in this setting [9].

In the present study, we focus exclusively on individuals who were receiving DTG-based therapy at the time of resistance testing, extending the observation period, and aim to further investigate the prevalence and patterns of resistance, describe mutational profiles, and explore clinical and programmatic implications in a larger and more comprehensive cohort.

## Methods

### Study design and setting

This is a retrospective observational study conducted across five centers of the DREAM program in Mozambique, including two centers in Maputo City, one in Maputo Province, and two in Sofala Province. The DREAM program is a community-based, primary care HIV initiative that integrates clinical follow-up, laboratory monitoring, and social support, and has been active in Mozambique since 2002 [10–13]. DREAM sites are health centers providing a wide range of services, including basic HIV care.

The current analysis builds on a previously published cohort [9], expanding the sample by including all eligible participants who underwent genotypic resistance testing due to confirmed virological failure, even after an adherence reinforce intervention, while on DTG-based ART between July 2022 and December 2024. Compared to the earlier analysis, the present study includes a broader time frame and applies slightly refined inclusion criteria, focusing exclusively on individuals who were receiving DTG at the time of resistance testing, to ensure a more consistent assessment of DTG-associated resistance.

### Study population

We included HIV-positive individuals on dolutegravir (DTG)-based antiretroviral therapy (ART) who experienced virological failure, defined as HIV RNA > 1000 copies/mL, between June 1, 2023, and December 31, 2024. According to DREAM protocols, individuals with virological failure were enrolled in a six-month enhanced adherence support program; no ART regimen changes are made during the enhanced adherence support phase. A repeated viral load test was performed after this period, and those with persistent viremia (> 1000 copies/mL) were eligible for genotypic resistance testing. Only individuals on DTG-containing regimens at the time of resistance testing were included in this analysis. Elsewhere we described the wider population of the individuals with elevated VL [9].

Inclusion criteria were: (i) confirmed HIV-positive status; (ii) being on a DTG-based regimen at the time of resistance testing; (iii) confirmed virological failure (HIV RNA > 1000 copies/mL) after at least six months of enhanced adherence support. Exclusion criteria included: (i) switching from DTG to another regimen before resistance testing; (ii) insufficient plasma samples for sequencing; or (iii) incomplete clinical records.

Due to the retrospective nature of the study and limitations in documentation, we were unable to determine the exact number of eligible individuals who did not undergo resistance testing. Genotypic testing was requested at the discretion of the treating clinicians, based on clinical judgment and operational feasibility. However, specific criteria guiding these decisions were not systematically recorded or analyzed. Logistical constraints such as reagent availability, sample transport, and staffing likely also contributed to the limited number of tests performed; hence, only a subset of eligible individuals underwent resistance testing.

### Resistance testing procedures

Resistance testing followed standardized DREAM protocols. Plasma samples from participants with confirmed virological failure were collected and sent to the Zimpeto DREAM laboratory, where viral RNA was extracted and amplified using an in-house reverse transcription and nested PCR method. Sequencing of the HIV integrase region was performed using the Sanger method at INQABA Biotechnical Industries, South Africa. Sequences were analyzed and interpreted directly at INQABA using the Stanford HIV Drug Resistance Database. DTG resistance was classified according to Stanford penalty scores as follows: susceptible (score < 10) (S), low-level resistance (10–14) (LLR), intermediate resistance (15–29) (IR), and high-level resistance (≥ 30) (HLR). Resistance mutations were identified in the integrase region, and, when available, in the reverse transcriptase and protease regions.

### Data collection and variables

Clinical and demographic data were extracted from the DREAM electronic medical records. Variables collected included age, sex, duration on ART, number of prior ART regimens, HIV-1 subtype (when available), and reason for initiating DTG (first-line, switch while suppressed, or switch after failure). Resistance to DTG was classified using Stanford scoring.

### Statistical analysis

Descriptive statistics were used to summarize baseline characteristics. Categorical variables were presented as frequencies and percentages, and continuous variables as medians with interquartile ranges (IQR).

### Ethics

The study was approved by the National Bioethics Committee for Health of Mozambique (Ref: 430/CNBS/24, July 19, 2024). Given the retrospective nature of the study and the use of routinely collected anonymized data, the requirement for informed consent was waived. The study was conducted in accordance with the principles of the Declaration of Helsinki and relevant national guidelines.

## Results

### Study population and baseline characteristics

In the considered period, 28 individuals on DTG-based ART who underwent resistance testing following confirmed virological failure (HIV RNA ≥ 1000 copies/mL) after six months of enhanced adherence support were included in the analysis. These represent all individuals for whom genotypic resistance test results were available, although additional eligible individuals did not receive testing due to logistical constraints. Among them, 13 (46.4%) had evidence of resistance to DTG, while 15 (53.6%) remained susceptible.

Table 1 summarizes clinical and demographic characteristics stratified by DTG susceptibility. Participants with DTG resistance were generally older (median age: 38 [IQR: 28–49] vs. 28 years [IQR: 18–40]), had longer treatment histories (median time on ART: 15 [IQR: 9–17] vs. 9 years [IQR: 6–13]), and were more frequently infected with HIV-1 subtype C (92.3% vs. 13.3%) compared to those without resistance. A history of multiple prior ART regimen changes was more common among individuals with DTG resistance: 61.5% (8 out of 13) of resistant individuals had been exposed to at least two previous ART regimens, compared to 40.0% (6 out of 15) of those without resistance. This suggests a greater cumulative risk of resistance development in those with more complex treatment histories. Resistance was observed across all categories of DTG initiation, including participants who started DTG while suppressed (46.2%) or as first-line therapy (7.7%). Among all the 28 participants, 3 received rifampicin-based anti-tuberculosis treatment during DTG treatment, all of them modified DTG dosage according to guidelines; one of them developed DTG resistance.

**Table 1:** Clinical and demographic characteristics of participants undergoing resistance testing, stratified by dolutegravir (DTG) susceptibility

Variable	Total ***participants*** tested (*n* = 28)	IQR/%	DTG sensible ***participants*** (*n* = 15)	IQR/%	DTG resistant ***participants*** (*n* = 13)	IQR/%
Age (median years, IQR)	34	22–43	28	18–40	38	28–49
Sex (n. %)						
M	10	35,71%	11	73,33%	9	69,23%
F	18	64,29%	4	26,67%	4	30,77%
Time in ART (median years, IQR)	11	8–15	9	6–13	15	9–17
HIV-1 subtype						
B	6	21,43%	5	33,33%	1	7,69%
C	14	50,00%	2	13,33%	12	92,31%
Unkonwn	8	28,57%	8	53,33%	0	0,00%
Reason to shift to DTG therapy						
In viral failure	13	46,43%	7	46,67%	6	46,15%
In viral suppression	11	39,29%	5	33,33%	6	46,15%
Start in DTG	3	10,71%	2	13,33%	1	7,69%
After discontinuation	1	3,57%	1	6,67%	0	0,00%
Previous regimen changes						
0	3	10,71%	2	13,33%	1	7,69%
1	11	39,29%	7	46,67%	4	30,77%
2	14	50,00%	6	40,00%	8	61,54%

### INSTI mutational profiles

Table 2 details the frequency of integrase strand transfer inhibitor (INSTI)-associated mutations among the 13 DTG-resistant individuals. The most common mutations were G118R and E138K, each detected in 7 participants (43.8%). These were followed by L74M (5 individuals, 31.3%) and R263K (4 individuals, 25.0%).

**Table 2:** Frequency of integrase strand transfer inhibitor (INSTI) mutations among participants with DTG resistance

Mutation	Occurrence	% of occurrence among the tested (multiple mutations possible)
G118R	7	43,75%
E138K	7	43,75%
*L74M*	5	31,25%
R263K	4	25,00%
G140A	2	12,50%
T66A	2	12,50%
*E157Q*	2	12,50%
E138A	1	6,25%
Q146P	1	6,25%
S147G	1	6,25%
Q148R	1	6,25%
Q148K	1	6,25%
T66I	1	6,25%
*S230SR*	1	6,25%
*G163R*	1	6,25%
*L74I*	1	6,25%

Several other mutations were also identified, including G140A, T66A, and E157Q (each in 2 participants, 12.5%), and a series of less frequent mutations—such as Q148R, Q148K, T66I, and S230SR—each observed in a single individual (6.25%).

### DTG resistance levels and co-resistance

**Fig. 1:**
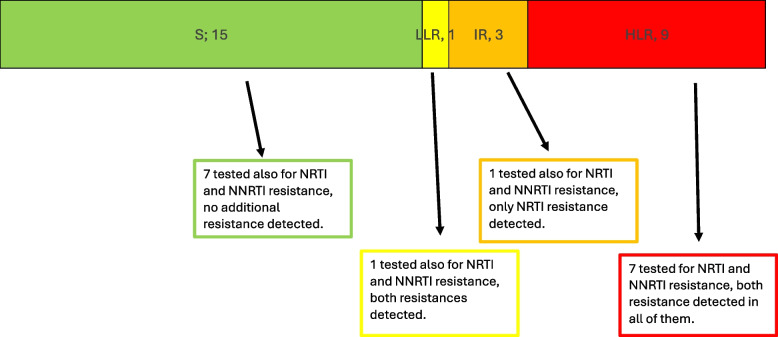
Distribution of DTG resistance levels according to Stanford classification (S = susceptible, LLR = low-level resistance, IR = intermediate resistance, HLR = high-level resistance). For each resistance category, results of concomitant NRTI and NNRTI resistance testing are shown where available (16 *participants* underwent testing for additional drug classes)

Figure 1 illustrates the distribution of DTG resistance levels and associated co-resistance. Based on Stanford interpretation, 1 of the 13 resistant participants had low-level resistance (LLR), 3 had intermediate resistance (IR), and 9 had high-level resistance (HLR). Among the 16 individuals tested for resistance to other antiretroviral classes, all were susceptible to protease inhibitors (PI). However, co-resistance was identified in all nine DTG-resistant individuals who underwent testing for other drug classes: eight were resistant to both nucleoside reverse transcriptase inhibitors (NRTIs) and non-nucleoside reverse transcriptase inhibitors (NNRTIs), and one to NRTIs only. The most frequent mutations were M184V and K65R, while no major PI resistance mutations were detected. The burden of co-resistance increased with the severity of DTG resistance, with multi-drug resistance for NRTI and NNRTI predominantly observed among those with HLR.

### Resistance by treatment history

**Fig. 2:**
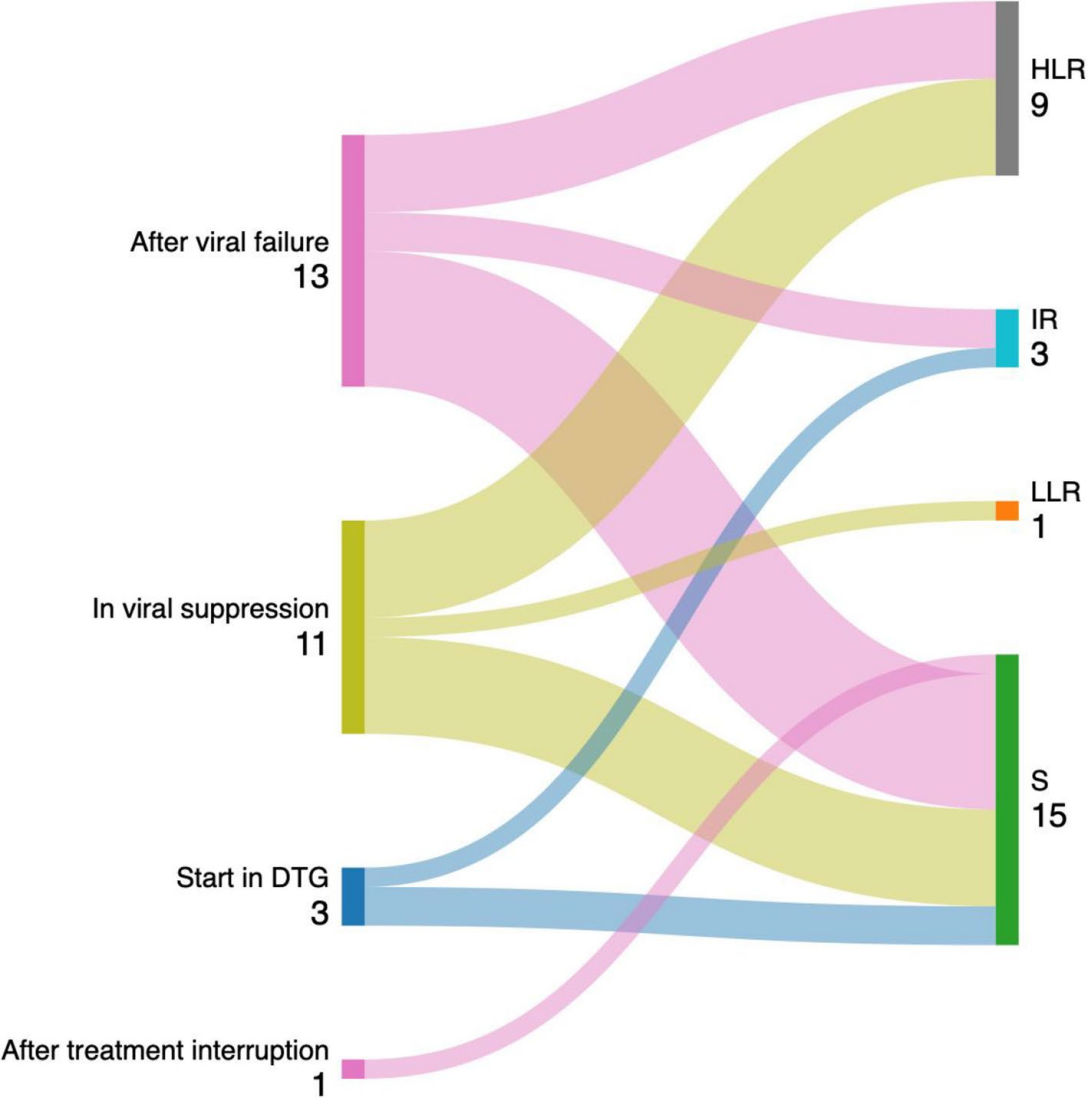
Sankey diagram illustrating the relationship between reason for DTG initiation and level of resistance to DTG

Figure 2 presents a Sankey diagram that visualizes the relationship between the reason for initiating DTG and the level of resistance detected in genotypic testing. The diagram shows that HLR emerged across all three groups. Specifically, HLR was identified in individuals who had switched from a failing regimen, but also in participants who started DTG while virologically suppressed and even in those who began DTG as their first-line regimen.

## Discussion

The results of our study, conducted in five DREAM program centers in Mozambique between 2023 and 2024, reveal the presence of mutations associated with resistance to DTG among individuals with virological failure. Specifically, 42.8% (12 out of 28) of the participants tested for resistance presented integrase-region mutations compatible with intermediate or high level resistance to DTG. Despite the great effectiveness of DTG-based regimens, emerging African literature is documenting an increasing incidence of DTG resistance, particularly in real-world settings, contrasting with data from controlled clinical trials [3]. In our previous study, we reported a suppression control rate among patient on DTG-based treatment very high (95%), which clearly demonstrates the success of such regimens; however, a growing body of literature is showing also the potential risk of the emergence of DTG-resistance in the African continent. Although the percentage of resistance remains relatively low, it could still translate into thousands of affected patients across the region.

In large randomized studies such as the NADIA trial (Uganda, Kenya, Zimbabwe) and the DAWNING trial, the rate of INSTI resistance emergence remained low (4% and 1%, respectively), thanks to intensive viral load monitoring and optimized therapeutic strategies [14, 15]. However, these conditions are rarely replicated in routine African healthcare contexts, where virological failures may go undetected for a long period of time. In real-world cohorts similar to ours, such as that reported by Ismael et al. in Mozambique, the prevalence of INSTI mutations among individuals with DTG failure reached 15–20% [16]; in other cohorts, with different selection criteria, the rates where even higher [17]. While these findings are concerning, current evidence does not allow us to determine whether the incidence of DTG resistance is truly increasing over time, or simply more frequently identified as resistance testing becomes more accessible and widely implemented.

The G118R and E138K mutations, each found in 43.8% of our resistant cases, are among the most frequently reported DTG resistance pathways in non-B subtypes, particularly subtype C, which was predominant in our sample. The relevance of G118R has been highlighted by several studies: the meta-analysis by Semengue et al. identified G118R and R263K as the most common INSTI mutations in African failures [7], while the review by Kamori and Barabona emphasized its selection in subtypes A and C, facilitated by its low fitness cost [5]. The G118R + R263K combination, also observed in our study, has been associated with high-level resistance and reduced viral fitness [18], and biochemical studies [19] confirmed its preferential selection in subtype C.

Our study also reinforces the strong association between a complex treatment history and resistance risk. DTG-resistant participants had a significantly longer treatment history (median 15 years) and more frequent past failures. This is consistent with Loosli et al., who reported similar patterns in a large African-European cohort [6]. Furthermore, we observed INSTI mutations even in participants who were DTG-naïve or had initiated the drug while virologically suppressed. This finding are in line with reports from Mahomed et al., Fourie et al., and Mens et al., where G118R emerged under conditions of suboptimal adherence or functionally ineffective regimens [20–22].

The observed co-resistance patterns—particularly the frequent presence of M184V and K65R—highlight the threat of multi-drug resistance in individuals failing DTG-based regimens, even in the absence of PI resistance. This pattern mirrors findings from other papers who reported NNRTI and NRTI resistance in participants failing DTG-based therapy [16, 17]. Our results therefore reinforce the concern that switching to DTG without assessing virological suppression may lead to resistance selection on a background of already compromised backbones.

Lastly, our findings take on special relevance considering the growing interest in long-acting therapies such as cabotegravir. The G118R mutation, also observed in our study, has been associated with reduced susceptibility to cabotegravir in vitro, raising concerns about cross-resistance [5, 23]. The editorial by Fokam et al. in Nature Medicine strongly advocates for integrating resistance testing into routine clinical care in low-income countries to avoid silent accumulation of INSTI mutations. Similarly, the review by Tao et al. emphasizes that persistent, unrecognized viremia is a key driver of resistance emergence even in ART-naïve individuals, a situation reflected in our cohort [24].

### Limitations

This study has several limitations. First, due to the small number of participants included (n = 28) and the fact that genotypic resistance testing was not performed systematically on all individuals with virological failure, the epidemiological value of our prevalence estimates is limited.

Second, resistance testing was restricted to the integrase region only for about half of the samples, additional mutations in other regions (e.g., protease or reverse transcriptase) might have gone undetected in participants not fully sequenced. Moreover, Stanford scores were available only for a subset of individuals, precluding systematic classification of resistance levels across the full sample.

Finally, data on viral load trajectories, adherence, and treatment history were incomplete for some participants, limiting the capacity to reconstruct the exact pathway leading to the emergence of resistance in each case. Despite these limitations, our study provides important early signals on the possible patterns of DTG resistance in real-world Mozambican settings and highlights the urgent need for expanded molecular surveillance.

### Public Health Implications

The findings of this study could have implications for public health policy and HIV program implementation in sub-Saharan Africa. First, the emergence of DTG resistance—even among participants initiating treatment while virally suppressed—highlights the need to revise current "test-and-switch" strategies. We are aware of the effectiveness of DTG-based regimens, but we argue that virological monitoring and resistance testing should be strengthened prior to transitioning individuals to DTG-based regimens, especially in settings with a history of limited adherence or prior treatment failures. In the actual guidelines, WHO recommends the availability of genotyping testing only in context in which DTG-based regimens are not available; our results, together with other similar data, suggest the need to revise this recommendation, and make resistance testing more available on the field, in Africa, too.

For these reasons, our data underscore the urgency of integrating genotypic resistance testing into national HIV guidelines and decentralizing access to such diagnostics. Establishing sentinel surveillance systems in high-prevalence areas could allow for early detection of resistance trends and inform timely changes in treatment protocols.

Finally, the potential for cross-resistance to newer long-acting therapies such as cabotegravir calls for cautious and evidence-based scale-up of such interventions, considering also the extensive resistance to NNRTI typically present in individuals having failed first generation NNRTI regimens [3], that might affect the efficacy of rilpivirine (the second drug present in the long-acting regimen). Investments in molecular diagnostic capacity and targeted training of healthcare workers are essential to ensure that therapeutic innovations do not inadvertently accelerate resistance at the population level.

In summary, our data confirm and expand on recent African evidence regarding DTG resistance: G118R, E138K, R263K, and L74M represent emerging mutational patterns in Mozambique, particularly among individuals with extensive treatment history, unsuppressed viral load, or prior failures. The possible spread of such mutations, even in clinically stable individuals, may have implications for the long-term sustainability of DTG-based regimens and highlights the need to implement sentinel genotypic surveillance strategies in public treatment programs.

## Conclusion

This retrospective analysis describes the presence of integrase inhibitor resistance mutations among individuals with virological failure on DTG-based ART in five DREAM centers in Mozambique. Among those tested, nearly half showed evidence of resistance, with a predominance of G118R and other mutations previously associated with DTG failure in non-B subtypes. The findings reflect the need for ongoing monitoring of DTG resistance in routine programmatic settings and support the value of implementing targeted genotypic surveillance where feasible. Further studies with systematic sampling will be important to better estimate the prevalence and clinical implications of resistance in similar contexts.

## Data Availability

The datasets used and/or analyzed during the current study are available from the corresponding author on reasonable request.
